# Season, Microclimate and Shoreline Disturbance Interactively Shape Bird Functional Diversity

**DOI:** 10.1002/ece3.72901

**Published:** 2026-01-07

**Authors:** Samuel E. Tamekloe, Joseph K. Daniels, Kweku A. Monney, Justus P. Deikumah

**Affiliations:** ^1^ Department of Conservation Biology and Entomology, School of Biological Sciences, College of Agriculture and Natural Sciences University of Cape Coast Cape Coast Ghana

**Keywords:** conservation, functional diversity, seasonal dynamics, tropical coastal ecosystems, urbanization, West Africa

## Abstract

Understanding how ecological processes shape avian community structures remains fundamental to conservation in tropical coastal ecosystems, where birds serve crucial roles as seed dispersers, scavengers, and prey population regulators. West African coastal habitats face mounting pressures from urbanization and changing microclimate; however, we lack a comprehensive understanding of how these stressors interact with seasonal cycles to influence functional diversity. We examined how seasonality, microclimate, and shoreline disturbance shape bird functional diversity along a 15 km coastal transect in Ghana's Central Region. Bird communities were surveyed across the dry and wet seasons at 30 points. Four functional diversity indices (Functional Richness, Evenness, Divergence, and Dispersion) were quantified using trait‐based approaches. Multivariate analyses demonstrated that the interaction between the shoreline condition and season significantly influenced trait composition (PERMANOVA: *R* ^2^ = 0.045, *p* < 0.001), with natural shorelines exhibiting pronounced seasonal functional turnover that was suppressed in urbanized sites. Natural shorelines showed higher functional dispersion during the dry season (4.01 ± 0.29) than the wet season (1.90 ± 0.16), whereas urbanized shorelines maintained stability year‐round, dominated by generalists, including Pied Crow ( *Corvus albus* ) and Yellow‐billed Kite ( *Milvus aegyptius* ). Functional divergence increased from the dry to wet seasons (0.877–0.901), while functional richness was higher during the wet seasons (6.72 vs. 5.29). Cloud cover was the most consistent environmental predictor, negatively associated with functional dispersion (*β* = −0.012, *p* < 0.001) but positively associated with divergence (*β* = 0.004, *p* < 0.01). Air temperature *β* = 0.316, *p* < 0.001 and low tidal levels (*β* = 0.318, *p* < 0.05) positively influenced dispersion. These findings demonstrate that urbanization dampens the temporal dynamics essential for ecosystem resilience, emphasizing the need to preserve natural shoreline integrity for maintaining seasonal functional turnover in tropical coastal regions.

## Graphical abstract

This study examined the seasonal and spatial patterns of bird functional diversity across natural and urbanized coastal sites in Ghana. We found that natural shorelines undergo significant seasonal functional reorganization, whereas urbanized areas maintain stable, generalist‐dominated communities year‐round, losing the temporal dynamics that support ecosystem resilience. Preserving the integrity of natural shorelines is therefore critical for maintaining seasonal functional turnover and ecological responsiveness along the rapid developing West African coasts.

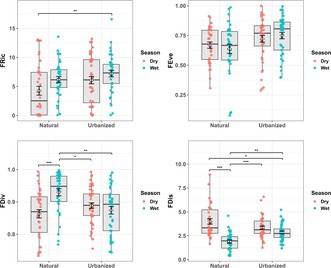

## 1 Introduction

Birds play essential ecological roles in nearly every ecosystem, contributing to processes such as seed dispersal, pollination, scavenging, and regulating prey populations (Whelan et al. 2008; Sekercioglu et al. 2019). Their mobility and well‐documented functional traits have long made them reliable indicators of environmental change (Gregory et al. 2005). In coastal ecosystems, birds contribute to ecological processes such as nutrient cycling, plant regeneration, and the maintenance of trophic balance. Coastal regions support diverse resident and migratory bird populations and are recognized as biodiversity hotspots (Barbier et al. 2011; Ntiamoa‐Baidu et al. 2014). These vital habitats are increasingly becoming vulnerable to anthropogenic stressors, including rapid urbanization, shoreline modification, and shifting climatic patterns (Lotze et al. 2006; Nicholls et al. 2007).

The structure and heterogeneity of habitats are key determinants of bird community composition (Stein et al. 2014; Cooper 2023). Complex and minimally disturbed environments generally support greater functional diversity (Rodrigues et al. 2009), whereas environmental simplification caused by the development or vegetation loss reduces trait variability and favors generalist species (Devictor et al. 2008; Clavel et al. 2011; Rubeena et al. 2023). Along West African tropical coasts, including the Ghanaian shoreline where this study was conducted, natural habitats form a mosaic of mangroves, mudflats, sandy beaches, and littoral thickets that provide diverse nesting and foraging resources for resident and migratory birds. Seasonal rainfall drives pronounced shifts in vegetation cover, prey availability, and water levels, which can restructure functional traits through changes in trophic ecology, body size distributions, and dispersal capacity. Coastal urbanization and land reclamation have fragmented and simplified these habitats through the replacement of natural substrates with built structures and vegetation clearance along the West African region (Sengupta et al. 2023). The Ghanaian coast is not spared from these pressures, experiencing rapid urbanization, extensive sand mining operations that destabilize shoreline geomorphology, and accelerating land conversion that continues to erode the extent and connectivity of natural coastal habitats (Kutir et al. 2022; Kankam et al. 2025). Therefore, we expect that natural coastal segments should show stronger seasonal restructuring of functional traits, while urbanized areas should exhibit reduced trait turnover and a dominance of generalist or disturbance‐tolerant species.

While the functional diversity of tropical birds has been examined in Amazonian forests (Bregman et al. 2016) and agroecological systems in Argentina (Lorenzón et al. 2020), as well as in other tropical regions, coastal assemblages remain largely overlooked despite their ecological importance and exposure to human disturbance. In Africa, studies have begun to apply trait‐based approaches, including works in rainforest and savannah systems in Nigeria (Awoyemi et al. 2024), multimetric assessments using African Bird Atlas data (Lee et al. 2021), and investigations of savannah bird communities in northern Ghana (Sulemana et al. 2022), but none focus on tropical coastal bird communities. Thus, the influence of combined gradients of seasonality, microclimate, and shoreline modification (Keyser et al. 2024; Aarif et al. 2025) remains untested in West Africa.

The use of functional diversity metrics is particularly relevant because taxonomic diversity alone provides limited insight into ecosystem functioning; species richness does not necessarily imply functional complementarity. Functional diversity, derived from traits linked to resource use and ecological roles, reveals how communities sustain key processes such as pollination, seed dispersal, and nutrient cycling (Wenny et al. 2011; Sekercioglu et al. 2019). In coastal habitats exposed to urban development and seasonal variability, such trait‐based metrics are especially valuable for understanding ecosystem resilience. We therefore employed four complementary indices of functional diversity that capture distinct aspects of community trait structure (Villéger et al. 2008; Mouillot et al. 2013). Functional richness (FRic) quantifies the range of trait space occupied by species, reflecting the potential breadth of ecological functions. Functional evenness (FEve) measures the regularity of species distributions within that trait space, indicating how efficiently available niches are filled. Functional divergence (FDiv) describes how traits are distributed toward the extremes of functional space, representing niche differentiation and resource partitioning. Finally, functional dispersion (FDis) integrates trait distances weighted by abundance, providing an overall measure of community‐level trait spread and redundancy. Together, these indices allow us to identify complementary and contrasting patterns of functional organization that a single diversity measure could overlook.

This study forms part of a broader project investigating how environmental gradients shape bird functional diversity in tropical coastal ecosystems, using Ghana as a model for rapidly urbanizing West African seascapes. Traits were selected to capture key ecological axes, including trophic ecology, foraging behavior, foraging strata, migratory tendency, habitat preference, average body weight, average body length, and average wingspan. We focused on how seasonality, microclimatic variation, and shoreline disturbance could be associated with our four functional diversity indices: FRic, FEve, FDiv, and FDis. In the study area, rainfall seasonality is closely linked to shifts in the vegetation structure, prey abundance, and water levels, while bird migration adds a second layer of temporal turnover (Ntiamoa‐Baidu 1991). Peak arrival of long‐distance migratory species coincides with the dry season, when inland habitats become less favorable, whereas the onset of the rains marks the early arrival and late departure of several Palearctic and intra‐African migrants. These overlapping climatic and migratory cycles are expected to restructure functional traits directly tied to resource acquisition and movement capacity.

We test the following four hypotheses: (1) functional diversity will decline during the dry season as resource availability contracts and competition intensifies; (2) natural shorelines will exhibit higher FRic and FDiv than urbanized ones, reflecting broader niche complementarity and greater resource heterogeneity; (3) the interaction between the shoreline condition and season will modulate trait turnover, such that natural habitats show stronger seasonal restructuring of functional composition, whereas urbanized sites, being structurally simplified and resource‐limited, will exhibit reduced seasonal variation in functional traits; (4) sites with greater microclimatic variability will support higher FDis by accommodating a wider range of foraging strategies.

Beyond its regional importance, this study contributes to the global understanding of how tropical ecosystems, particularly coastal zones, mediate biodiversity responses to increasing human pressures. As tropical coastlines experience accelerating habitat transformation, our findings offer a unique perspective into how ecological functions linked to avian communities respond to changing environmental conditions. By linking trait‐based diversity patterns to microclimatic and disturbance gradients, this study advances understanding of functional resilience and ecological filtering in tropical coastal bird communities. These findings address a critical gap in tropical coastal conservation science and provide a foundation for long‐term ecological monitoring, habitat restoration, and climate‐resilient conservation planning in West Africa.

## 2 Materials and Methods

### 2.1 Study Area

**FIGURE 1:**
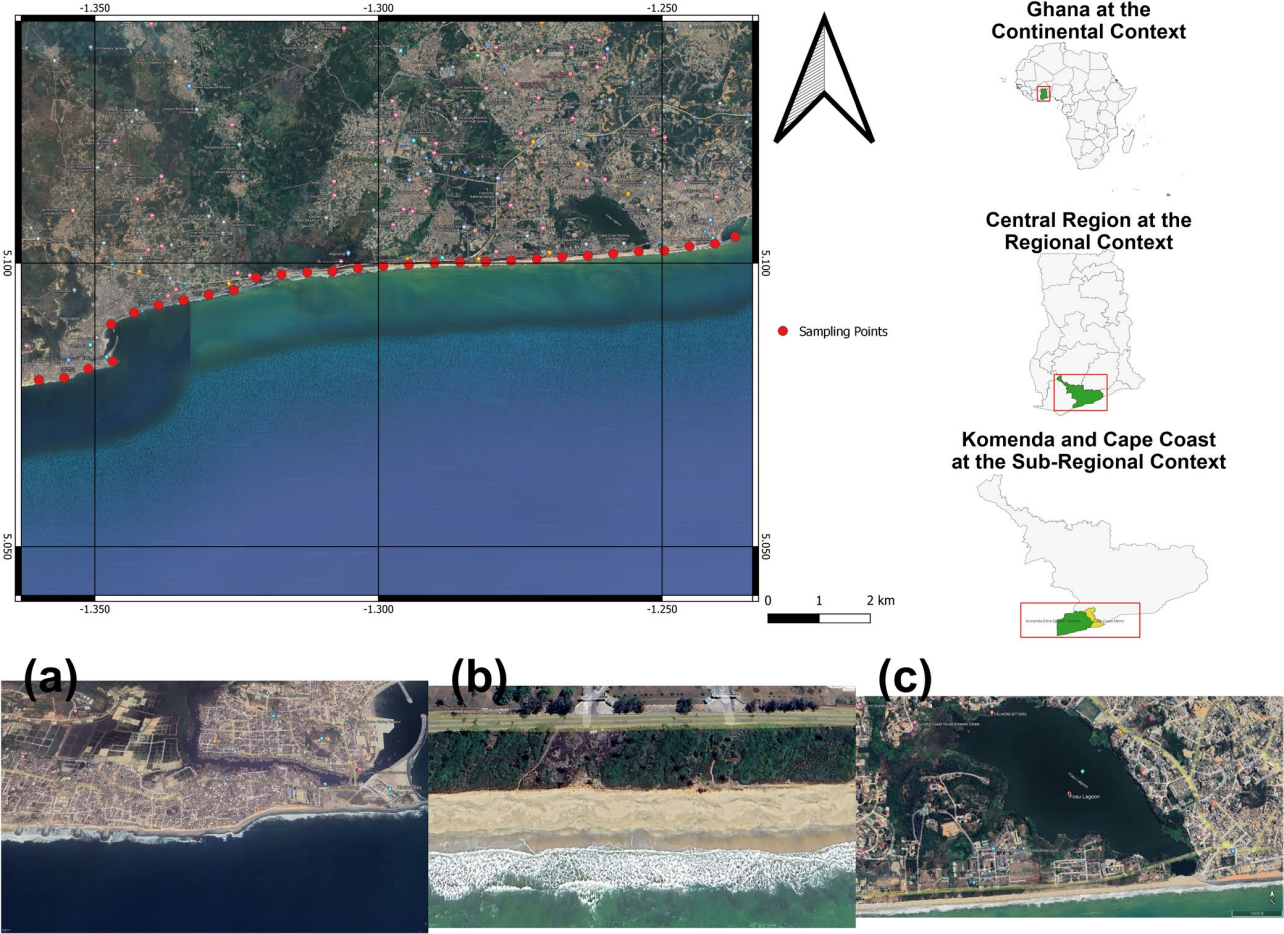
Study site locations along the Central Region coastline of Ghana, showing sampling points across different shoreline types. The map depicts the distribution of survey sites categorized by some structural factors: (a) heavily urbanized coastal areas with infrastructure development, (b) naturally vegetated coastal regions, and (c) coastal areas adjacent to fringe lagoons.

The study area is part of the 550‐km Ghana coastline that lies within two major migratory flyways (East Atlantic and Mediterranean). This coastline is important as it serves as feeding, roosting, and nesting grounds, especially for migratory birds (Smit and Piersma 1989; Gbogbo and Attuquayefio 2010; Borrow 2020). The coastal vegetation of Ghana includes mangrove stands, coastal scrub, and thicket, with some tropical forest remnants (Ayensu et al. 1996). The intertidal zones contain mudflats, lagoons, sandy beaches, and rocky outcrops (Entsua‐Mensah et al. 2000). The study was conducted along a 15‐km transect extending between the coasts of the Cape Coast Metropolitan (5.1732° N, 1.2987° W) and the Komenda‐Edina‐Eguafo‐Abirem Municipal (5.1207° N, 1.4628° W) Assemblies in Ghana's Central Region (Figure 1). The transect encompassed predominantly sandy substrate (50% of sampling points) and armored shoreline (43%), with representation of wooded coastal vegetation (30%), human settlements (53%), and smaller proportions of lagoon, estuarine, and rocky habitats. The Cape Coast–Elmina stretch was selected as a representative and accessible pilot site within a broader national project. This section combines diverse shoreline types (urbanized and seminatural), high bird activity, and strong human influence, offering a practical gradient for testing disturbance effects in this initial phase.

### 2.2 Study Design

This study was conducted between June 2023 and February 2024. We implemented a modified point‐count survey tailored to the structurally complex and heterogeneous coastal environment of southern Ghana. Thirty georeferenced observation stations were established along a 15‐km coastal transect, positioned approximately 15–20 m from the waterline and following the beach contour. This placement ensured that all stations remained within the active tidal influence, as water occasionally reached the observer's position during high tide. Each station covered a 100‐m observation radius, allowing the recording of birds using both the tidal zone and adjacent dune vegetation. Stations were spaced 500 m apart to minimize the likelihood of double‐counting individuals while maximizing spatial representation of bird assemblages (Bibby et al. 1998; Buckland et al. 2001). To further control for potential double‐counting and time‐of‐day bias, the starting point of each survey visit was alternated, beginning from the first station during one visit and from the last station during the next. Surveys were conducted during both high‐ and low‐tide periods to capture variation in bird activity across tidal phases, and all birds detected visually or aurally within the observation radius, including ground foraging, wading, and soaring species, were recorded. Sampling points were marked using handheld GPS devices (Garmin Etrex 10) to ensure consistent relocation across seasons. Birds were surveyed during morning (0600–0900 h) and evening (1500–1800 h) periods of peak activity. Each site was visited six times per season, consisting of three morning and three evening surveys paired as three complete rounds. To capture seasonal shifts in avian assemblages, surveys were conducted during the wet season (June–September), coinciding with peak breeding activity and early migrant arrival, and the dry season (December–February), when migratory species dominate, and residents adjust to reduced resource availability. At each station, a 10‐min observation period followed a 2‐min settling time to allow resumption of natural behavior (Verner 1985). A single experienced observer conducted all surveys to minimize interobserver variability. Incidental observations recorded while moving between points were excluded from quantitative analyses but were retained in a supplementary checklist. We acknowledge limitations inherent to this method, including potential underdetection of wide‐ranging or cryptic species such as raptors, which may be underrepresented in fixed‐duration counts (Bibby et al. 1998).

### 2.3 Functional Trait Classification

All recorded bird species were characterized using a comprehensive set of functional traits describing their trophic ecology, foraging behavior, foraging strata, migratory and movement patterns, habitat preference, and key morphological attributes. Dietary traits were coded in a binary (0/1) format to reflect the capacity of each species to consume multiple food resources, including insects and other small invertebrates, crustaceans, mollusks, annelids, fish, amphibians, small vertebrates, carrion, seeds, fruits, and nectar (Wilman et al. 2014; Şekercioğlu et al. 2025). Foraging behavior was similarly coded to represent multiple strategies per species, encompassing aerial sweeping, foliage gleaning, litter gleaning, bark gleaning, sallies, wading, pouncing, hawking, chasing and stabbing, seed or grain feeding, probing, diving, plunging, lunging, and scanning (Wilman et al. 2014; Sulemana et al. 2022; Mayani‐Parás et al. 2023). Foraging strata were also binary coded and defined as ground, understorey, midstorey, canopy, and surface water (Holbech 2009; Sulemana et al. 2022; Mayani‐Parás et al. 2023). Migratory and movement patterns were classified as residents, breeding migrants, partial intra‐African migrants, intra‐African migrants, Palearctic visitors, or Holarctic visitors (Borrow and Demey 2022). Habitat preference was assigned to the following categories: waterbody areas, agricultural areas, open savannah, woodland savannah, and human‐dominated areas (Deikumah et al. 2013; Şekercioğlu et al. 2025). Morphological traits included average body weight (g), average body length (cm), and wingspan (cm), obtained primarily from BIRDBASE, AVONET database, and Cornell Lab of Ornithology's Birds of the World (Tobias et al. 2022; Billerman et al. 2025; Şekercioğlu et al. 2025) and supplemented with regional field guides (Borrow 2020; Borrow and Demey 2022) where necessary. For a small number of species lacking wingspan data, we used body size‐based estimates or measurements from congeners within the same genus, which is an accepted practice when direct measurements are unavailable (Sheard et al. 2020).

### 2.4 Environmental Predictors and Habitat Classification

To quantify abiotic and structural factors potentially shaping avian functional diversity, we integrated both microclimatic and habitat‐level covariates across all survey points.

#### 2.4.1 Microclimatic Variables

Microclimatic variables were obtained from the Ghana Meteorological Agency's national network of automated weather stations, which provide high‐resolution, time‐stamped records of local atmospheric conditions. For each survey event, data on relative humidity, air temperature, cloud cover, and wind speed corresponding to the observation time were retrieved from the nearest operational station to the study transect. These measurements reflect the prevailing conditions during bird activity periods and thus represent time‐specific but spatially approximated estimates rather than direct on‐site readings. The variables were incorporated as continuous predictors in the general linear mixed models to evaluate their association with trait‐based functional diversity metrics.

#### 2.4.2 Shoreline Disturbance

Shoreline disturbance was operationalized as a binary variable distinguishing natural from urbanized coastal systems. Sites classified as natural were situated at least 150–200 m from the nearest built infrastructure and were surrounded primarily by vegetated dunes, lagoonal fringes, or estuarine zones with minimal direct human activity. In contrast, urbanized shorelines were located within 100 m of roads, buildings, or other human‐made structures, reflecting higher levels of anthropogenic disturbance. Classification was validated through field observations and geospatial inspection of satellite imagery to ensure consistent categorization across all sampling points.

### 2.5 Statistical Analysis

#### 2.5.1 Functional Diversity Computation

We quantified four multidimensional functional diversity indices: functional richness (FRic), functional evenness (FEve), functional divergence (FDiv), and functional dispersion (FDis), using the dbFD() function in the FD package (Laliberté and Legendre 2010). FRic represents the amount of functional trait space occupied by the community, reflecting the range of ecological strategies present. FEve quantifies the regularity with which species and their abundances fill that trait space, indicating how evenly functions are distributed. FDiv measures how species and their abundances diverge toward the extremes of trait space, signifying the degree of niche differentiation. FDis expresses the mean distance of individual species to the community centroid in trait space, capturing the overall dispersion of functional traits. Species‐by‐trait matrices constructed based on trophic ecology, foraging strata and behavior, migratory status, and habitat preference, average body weight, average body length, average wing span, and site‐level abundance were linked via unique replicate codes combining transect point, visit number, and season. Gower distances were used to accommodate mixed trait types, and principal coordinates analysis (PCoA) reduced trait dimensionality (Gower 1982), retaining only axes with positive eigenvalues.

#### 2.5.2 Testing Normality

**FIGURE A1:**
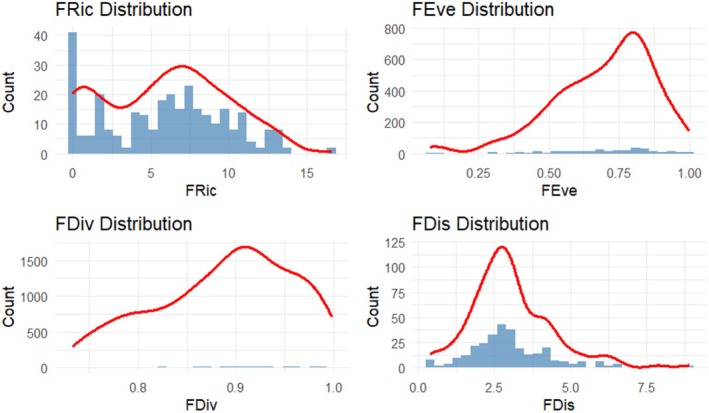
Visual distribution inspection.

Prior to analysis, we examined the distribution properties of each functional diversity metric through visual inspection (histograms) and Shapiro–Wilk normality tests (Appendix A: Figure A1, Table A1). All four metrics deviated significantly from normality (Shapiro–Wilk tests: all *p* < 0.001). Therefore, we employed distribution‐free approaches for community‐level comparisons (PERMANOVA, Kruskal–Wallis tests) and generalized linear mixed models (GLMMs) with appropriately specified error distributions for environmental driver analyses.

#### 2.5.3 Community‐Level Multivariate Analyses

We assessed differences in functional diversity using permutational multivariate analysis of variance (PERMANOVA) based on Gower dissimilarity matrices (Gower 1971) of four functional diversity metrics: functional richness (FRic), functional evenness (FEve), functional divergence (FDiv), and functional dispersion (FDis). The full model included shoreline conditions (natural vs. urbanized), seasons (dry vs. wet), and their interaction as fixed effects, with 4999 permutations (Anderson 2001). Pairwise PERMANOVA tests were conducted to identify specific group differences when main effects or interactions were significant.

For univariate analyses of individual metrics, we used Kruskal–Wallis tests to assess differences across groups, as preliminary analyses indicated non‐normal distributions for several metrics. Post hoc pairwise comparisons were performed using Dunn tests with Bonferroni correction for multiple comparisons (Dunn 1964). Effect sizes for univariate tests were calculated as eta‐squared (*η* ^2^) following Tomczak and Tomczak (2014), with magnitude classifications of small (0.01–0.06), moderate (0.06–0.14), and large (≥ 0.14) based on (Cohen 2013).

#### 2.5.4 Environmental Drivers: GLMMs

To identify environmental and temporal drivers of functional diversity, we fitted GLMMs for each functional diversity metric separately using the glmmTMB package (Brooks et al. 2017). For metrics bounded between 0 and 1 (FEve, FDiv), we applied beta regression with logit link functions following Douma and Weedon (2019), with values adjusted by a small constant (*ε* = 0.001) where necessary to avoid boundary issues. For unbounded, non‐negative metrics (FRic, FDis), we fitted Gaussian models with identity link functions after confirming acceptable residual distributions. Final distribution selection was based on a combination of Akaike Information Criterion (AIC) values and visual inspection of residual diagnostics using the DHARMa package (Hartig 2016), prioritizing models with minimal assumption violations over strict AIC minimization.

Fixed effects included time of day (morning vs. evening), humidity (%), cloud cover (%), wind speed (km/h), air temperature (°C), and tidal level (high vs. low). Site identity (points) was included as a random intercept to account for repeated sampling and spatial nonindependence (Zuur et al. 2009). Prior to modeling, we assessed multicollinearity among predictors using variance inflation factors (VIF; Fox et al. 2007). Model residuals were evaluated using DHARMa simulations (Hartig 2016), which provided standardized residuals through simulation from the fitted model, enabling robust assessment of goodness of fit, dispersion, and outliers regardless of the underlying distribution family. Spatial autocorrelation in model residuals was assessed using Moran's I (Moran 1950), calculated on residuals aggregated by site coordinates using a k‐nearest‐neighbors spatial weight matrix (Bivand and Wong 2018).

We evaluated model fit using marginal *R* ^2^ (variance explained by fixed effects) and conditional *R* ^2^ (total variance explained) following Nakagawa and Schielzeth (2013). Marginal effects of significant predictors were extracted and visualized using the ggeffects package (Lüdecke 2018). For categorical predictors, predicted means and 95% confidence intervals were obtained using estimated marginal means (emmeans package; Lenth 2023). Statistical significance was set at *α* = 0.05.

#### 2.5.5 Software and Packages

All analyses were conducted in R version 4.5.1 (R Core Team 2016) using the packages vegan (Oksanen et al. 2007), adonis for PERMANOVA (Li et al. 2022), cluster (Maechler 2018) for dissimilarity calculations along with packages like FD (Laliberté et al. 2014), FSA (Ogle and Ogle 2017) for Dunn tests, rstatix (Kassambara 2019) for effect size calculations, glmmTMB (Magnusson et al. 2017) for mixed models, DHARMa (Hartig and Hartig 2017) for residual diagnostics, performance (Lüdecke et al. 2021) for model *R* ^2^, car (Fox et al. 2007) for VIF, spdep (Bivand et al. 2017) for spatial autocorrelation, ggeffects (Lüdecke 2018) for marginal effects, emmeans (Lenth 2023) for estimated marginal means, ggplot2 (Wickham et al. 2016), and patchwork (Pedersen 2019) for composite visualizations.

## 3 Results

### 3.1 Seasonal Variation in Bird Functional Diversity

Functional diversity varied markedly between the dry and wet seasons (Global PERMANOVA: *F* = 10.49, *R* ^2^ = 0.063, *p* < 001, Table 1), indicating that seasonal dynamics strongly influenced community trait composition. Pairwise PERMANOVA confirmed the significant contrast between seasons (*F* = 9.88, *R* ^2^ = 0.063, *p* < 0.001).

**TABLE 1:** Permutational multivariate analysis of variance (PERMANOVA) for associations among the season, shoreline state, and their interaction with bird functional diversity.

Factor	*F*	*R* ^2^	*p*
Season	10.492	0.063	< 0.001***
shoreline state	4.476	0.027	< 0.01**
Season × shoreline state	7.548	0.045	< 0.001***

*Note:* Significance levels are indicated as follows: *** *p* < 0.001; **p < 0.01.

**FIGURE 2:**
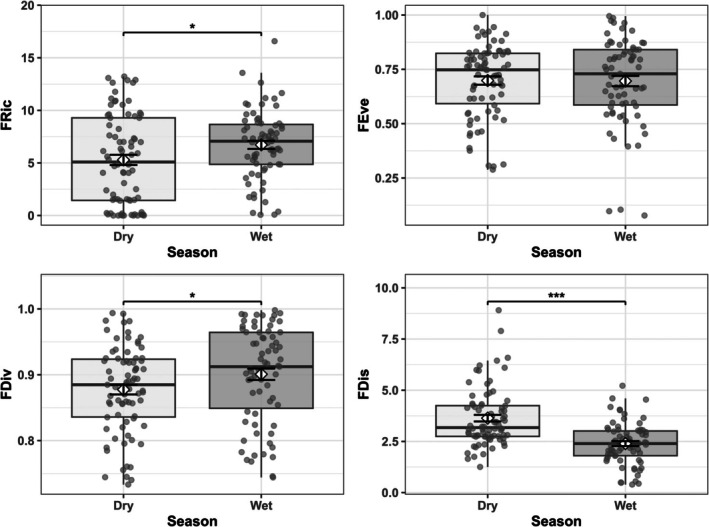
Functional diversity differs between the dry and wet seasons in dispersion, divergence, and richness but not in evenness. Box plots compare functional diversity metrics between the dry and wet seasons, pooled across shoreline types: functional richness (FRic), functional evenness (FEve), functional divergence (FDiv), and functional dispersion (FDis). Box plots display median (center line), interquartile range (box), 1.5× IQR (whiskers), and individual site values (gray points). Error bars represent mean ± SE. Asterisks denote significant differences (Dunn post hoc tests with Bonferroni correction: *p < 0.05, ***p < 0.001). Dry: *n* = 78; Wet: *n* = 71.

Seasonal shifts were most pronounced for functional dispersion, with a large effect size (*η* ^2^ = 0.215, Table 2) indicating substantial ecological restructuring (Figure 2 and Appendix B: Tables B1, B2, B3).

**TABLE 2:** Effect sizes for seasonal, shoreline state, and interaction effects on functional diversity metrics.

FD metric	Season (*η* ^2^)	Magnitude	Shoreline state (*η* ^2^)	Magnitude	Season × shoreline state (*η* ^2^)	Magnitude
FDis	0.215	Large	0.016	Small	0.292	Large
FDiv	0.029	Small	0.012	Small	0.112	Moderate
FEve	−0.006	Negligible	0.057	Small	0.048	Small
FRic	0.027	Small	0.028	Small	0.054	Small

*Note:* Significance levels are indicated as follows: ***p < 0.001; **p < 0.01; *p < 0.05; ns = not significant (*p* > 0.05).

Functional dispersion declined from the dry season (3.64 ± 0.16) to the wet season (2.39 ± 0.12; *p* < 0.001). Functional divergence increased from the dry to wet seasons (dry: 0.877 ± 0.007; wet: 0.901 ± 0.009; *p* < 0.05, *η* ^2^ = 0.029). Functional richness increased from 5.29 ± 0.49 in the dry season to 6.72 ± 0.39 in the wet season (*p* < 0.05, *η* ^2^ = 0.027). Functional evenness did not differ significantly between seasons (*p* > 0.05, *η* ^2^ = −0.006).

### 3.2 Shoreline Categorization Association With Bird Functional Diversity

Functional diversity also differed significantly between shoreline types (Global PERMANOVA: *F* = 4.476, *R* ^2^ = 0.027, *p* < 0.01, Table 1), with natural and urbanized shorelines supporting distinct functional assemblages (Pairwise PERMANOVA: *F* = 4.04, *R* ^2^ = 0.027, *p* < 0.05). This indicates that shoreline modification is associated with measurable restructuring of avian trait composition, but the moderate effect size (*R* ^2^ = 0.027) suggests that other environmental factors also shape functional structure.

**FIGURE 3:**
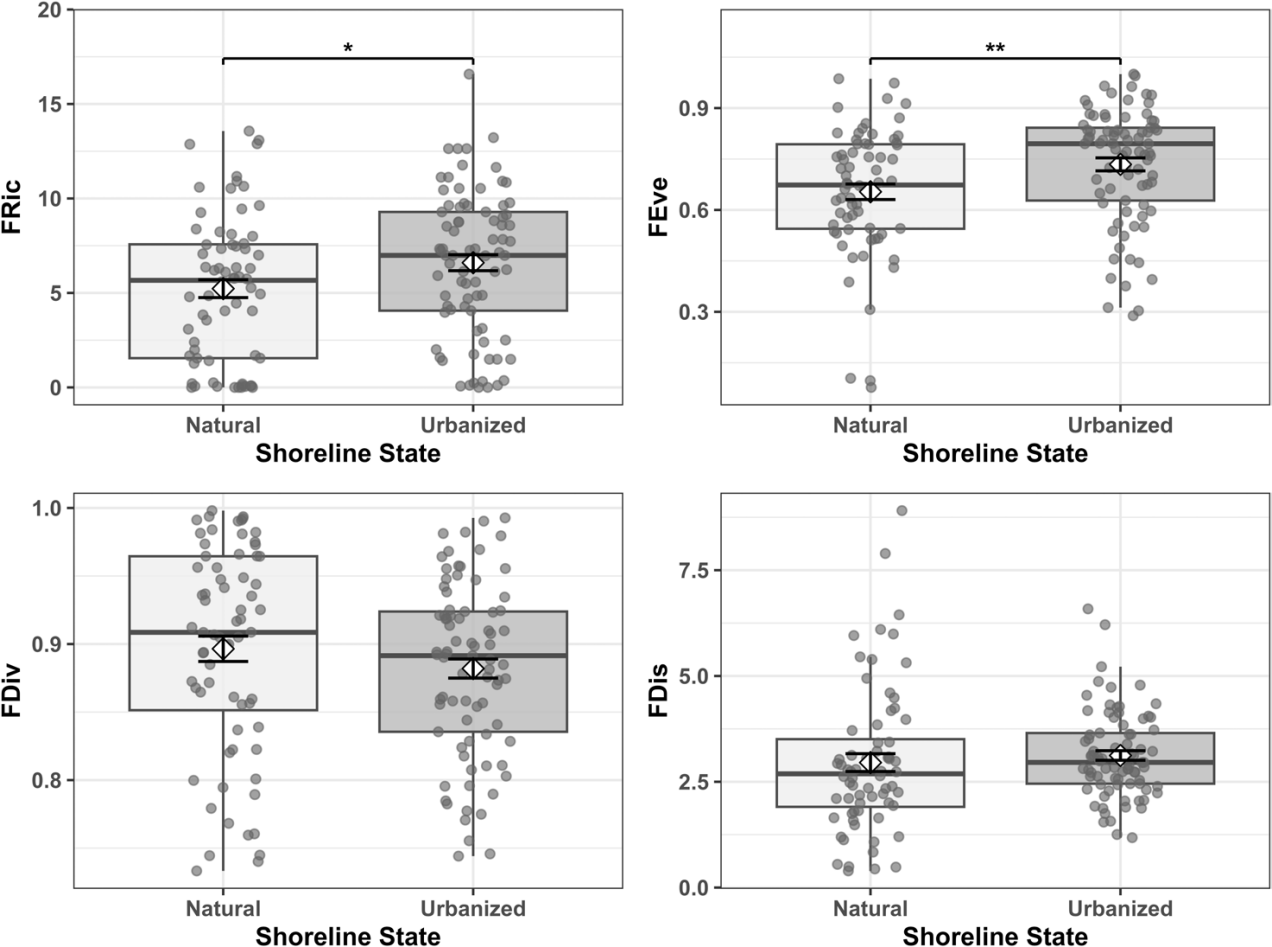
Functional diversity differs between the natural and urbanized shorelines in evenness and richness but not in dispersion or divergence. Box plots compare functional diversity metrics between the natural and urbanized shorelines: functional richness (FRic), functional evenness (FEve), functional divergence (FDiv), and functional dispersion (FDis). Box plots display median (center line), interquartile range (box), 1.5× IQR (whiskers), and individual site values (gray points). Error bars represent mean ± SE. Asterisks denote significant differences (Dunn post hoc tests with Bonferroni correction: *p < 0.05, **p < 0.01). Natural: *n* = 68; Urbanized: *n* = 81.

Univariate analyses revealed that urbanization primarily affected the distribution and breadth of functional space (Figure 3 and Appendix C: Tables C1, C2, C3). Functional evenness differed significantly between shoreline types (Kruskal–Wallis: *p* < 0.01, *η* ^2^ = 0.057), with urbanized sites exhibiting higher evenness (0.734 ± 0.019) than natural shores (0.653 ± 0.023), indicating a more uniform distribution of trait abundances in modified habitats. Functional richness also increased under urbanization (natural: 5.23 ± 0.47; urbanized: 6.60 ± 0.42; *p* = 0.024, *η* ^2^ = 0.028). However, functional dispersion (*p* = 0.066, *η* ^2^ = 0.016) and functional divergence (*p* = 0.100, *η* ^2^ = 0.012) showed no significant differences between shoreline types. Effect sizes were consistently small across all metrics (*η* ^2^ < 0.06).

### 3.3 Interactive Relationship Between Season and Shoreline Categories on Bird Functional Diversity

The interaction between the shoreline condition and season significantly influenced bird community composition (Global PERMANOVA: *F* = 7.55, *R* ^2^ = 0.045, *p* < 0.001, Table 1), indicating that the effects of shoreline modification vary across seasonal contexts. Pairwise comparisons (Table 3) revealed that bird assemblages on natural shorelines differed strongly between seasons (*R* ^2^ = 0.186, *p* < 0.001), reflecting pronounced seasonal turnover in undisturbed habitats. In contrast, urbanized shorelines showed no significant seasonal difference (*R* ^2^ = 0.020, *p* > 0.05), suggesting that seasonal restructuring of functional traits, prominent in natural habitats, is largely suppressed under urban influence.

**TABLE 3:** Pairwise PERMANOVA comparison for shoreline × season interaction effects on functional diversity.

Group1	Group2	*F*	*R* ^2^	*p*
Natural dry	Natural wet	15.127969	0.1864704	< 0.001***
Natural dry	Urbanized dry	3.504673	0.0440814	< 0.05*
Natural dry	Urbanized wet	6.558684	0.0868025	< 0.01**
Natural wet	Urbanized dry	10.075179	0.1170509	< 0.001***
Natural wet	Urbanized wet	9.263027	0.1183576	< 0.001***
Urbanized dry	Urbanized wet	1.580064	0.0196086	> 0.05ns

*Note:* Significance levels are indicated as follows: ***p < 0.001; **p < 0.01; *p < 0.05; ns = not significant (*p* > 0.05).

**FIGURE 4:**
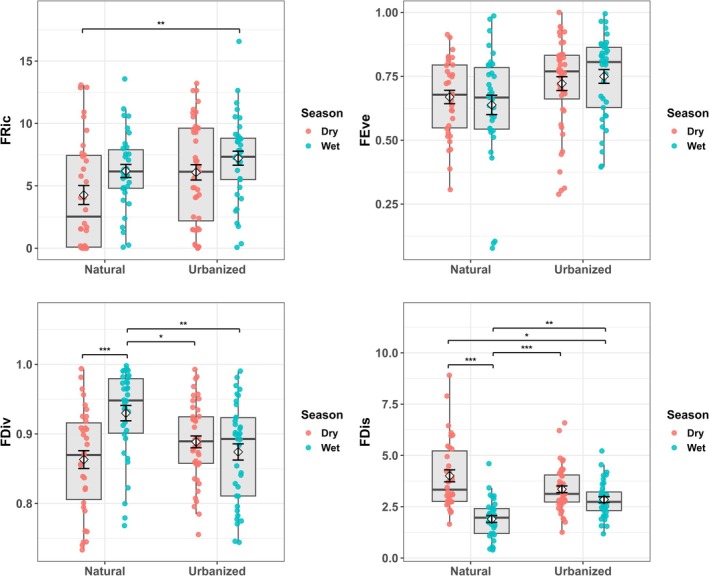
Seasonal variation in functional diversity differs between the natural and urbanized shorelines. Box plots show median (center line), interquartile range (box), 1.5× IQR (whiskers), and individual site values (points) for functional richness (FRic), functional evenness (FEve), functional divergence (FDiv), and functional dispersion (FDis) across shoreline types and seasons. Dry season samples are shown in coral and wet season samples in teal. Error bars represent mean ± SE. Asterisks denote significant pairwise differences (Dunn post hoc tests with Bonferroni correction: *p < 0.05, **p < 0.01, ***p < 0.001). Sample sizes: natural dry *n* = 34, natural wet *n* = 34, urbanized dry *n* = 44, and urbanized wet *n* = 37.

This interaction manifested differently across functional diversity dimensions. The effect was large for functional dispersion (FDis: *η* ^2^ = 0.292) and moderate for functional divergence (FDiv: *η* ^2^ = 0.112), but relatively small for functional richness and evenness (*η* ^2^ < 0.06; Table 2). Natural shorelines exhibited higher functional dispersion during the dry season (4.01 ± 0.29) than the wet season (1.90 ± 0.16), a seasonal pattern absent in urbanized sites (Figure 4). Functional divergence increased seasonally only in natural habitats (dry: 0.863 ± 0.013; wet: 0.930 ± 0.011), with wet‐season natural communities supporting more functionally extreme assemblages than urbanized sites in either season (Figure 4 and Appendix D: Tables D1, D2, D3).

Examination of species composition revealed possible mechanisms driving these observed functional diversity patterns (Appendix D: Tables D4, D5). Urbanized sites were dominated by large‐bodied (550–855 g), omnivorous/carnivorous species, particularly Pied Crow ( *Corvus albus* ) and Yellow‐billed Kite ( *Milvus aegyptius* ), which showed consistent abundances across seasons. Natural shorelines exhibited greater seasonal turnover, with smaller‐bodied specialists (25–40 g) driving compositional changes. The Village Weaver ( *Ploceus cucullatus* ), a woodland savannah resident, nearly doubled in abundance during the wet season (dry: 625; wet: 1205). During the dry season, the Common Sandpiper ( *Actitis hypoleucos* ), a Palearctic migrant, and the Black‐winged Stilt ( *Himantopus himantopus* ) were among the dominant species at natural sites.

### 3.4 Environmental and Temporal Correlates of Functional Diversity

**FIGURE 5:**
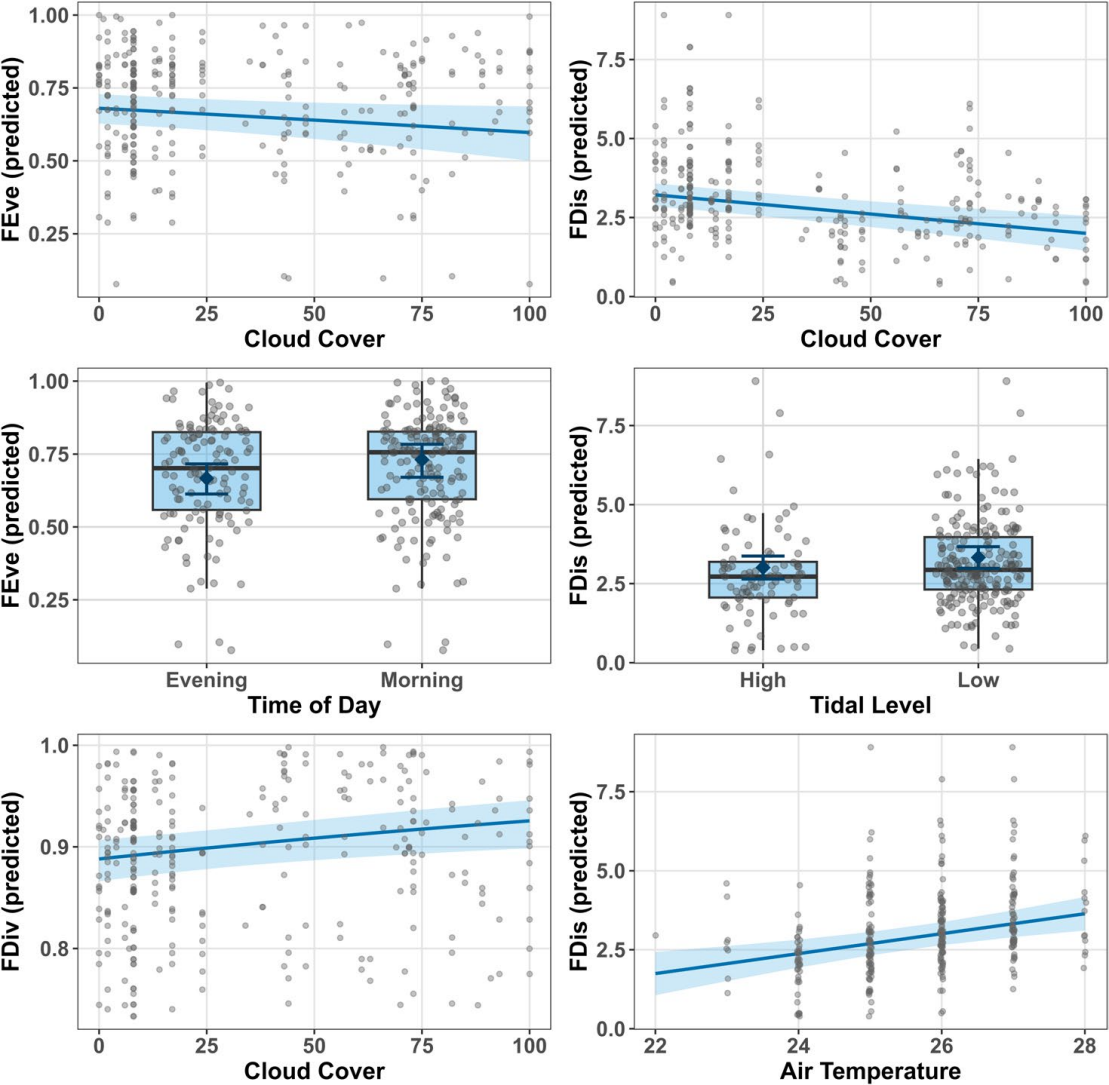
Relationships between functional diversity metrics and significant environmental predictors from generalized linear mixed‐effects models. Panels show predicted values (solid lines for continuous predictors, points for categorical predictors) with 95% confidence intervals (shaded ribbons or error bars). Individual observations (gray points) are jittered for visibility. Functional evenness (FEve) decreases with cloud cover. Functional dispersion (FDis) declines with cloud cover. Functional evenness differs between morning and evening observations. Functional dispersion increases at low tidal levels. Functional divergence (FDiv) increases with cloud cover. Functional dispersion increases with air temperature.

**FIGURE E1:**
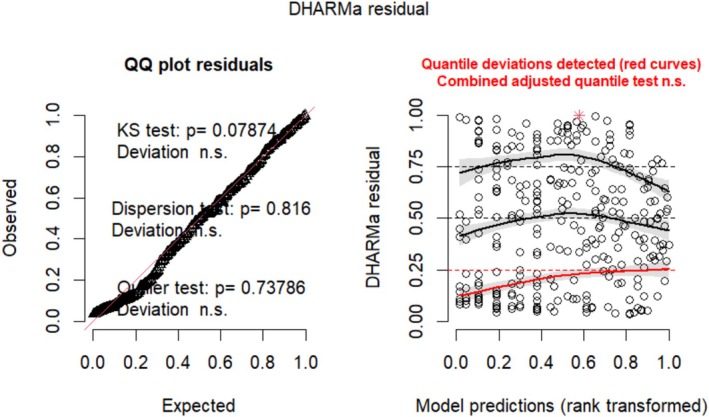
Diagnostic plot for the FRic model fitted with Gaussian regression log = “identity”.

**FIGURE E2:**
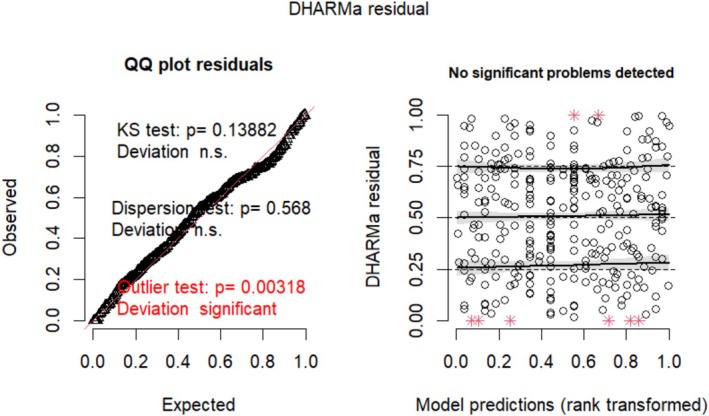
Diagnostic plot for FEve model fitted with beta regression link = “logit.”

**FIGURE E3:**
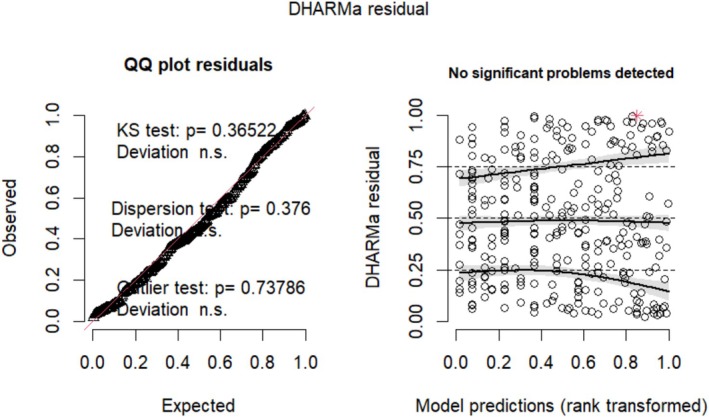
Diagnostic plot for FDiv model fitted with beta regression link = “logit”.

**FIGURE E4:**
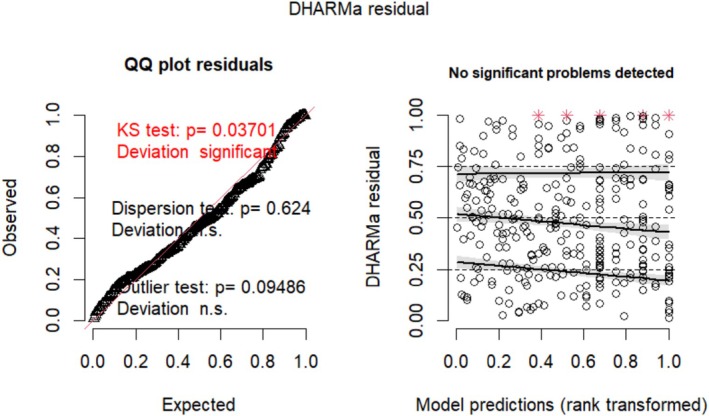
Diagnostic plot for the FDis model fitted with Gaussian regression log = “identity”.

GLMMs revealed that functional diversity metrics responded differently to environmental and temporal variables, with each model showing no major assumption violations (Figure 5; Appendix E: Figures E1, E2, E3, E4). Spatial autocorrelation in model residuals was negligible across all metrics (Moran's *I*: −1.53 to 0.13, all *p* > 0.44; Appendix E: Table E1), and multicollinearity among predictors was low (VIF < 3.3 for all variables; Appendix E: Table E2), validating model assumptions. Marginal *R* ^2^ values (proportion of variance explained by fixed effects) ranged from 0.037 (FRic) to 0.197 (FD is), while conditional *R* ^2^ values (total variance explained including random effects) ranged from 0.310 to 0.961, indicating substantial site‐level variation (Appendix E: Table E3).

Cloud cover emerged as the most consistent predictor, significantly associated with three of four functional diversity metrics, though with contrasting directionality. Functional dispersion declined with increasing cloud cover (*β* = −0.012, 95% CI: −0.017 to −0.008, *p* < 0.001), indicating that overcast conditions were associated with more functionally clustered assemblages. Functional evenness also decreased slightly with cloud cover (*β* = −0.004, 95% CI: −0.007 to 0.000, *p* < 0.05), suggesting reduced uniformity in trait abundance distributions under cloudier skies. Functional divergence increased with cloud cover (*β* = 0.004, 95% CI: 0.002–0.007, *p* < 0.01), indicating enhanced representation of extreme trait values. Functional richness showed no significant response to any measured environmental variable (all *p* > 0.05).

Air temperature and tidal state were specifically associated with functional dispersion. Warmer conditions were associated with greater trait dispersion (*β* = 0.316, 95% CI: 0.153–0.478, *p* < 0.001), as were low tidal levels (*β* = 0.318, 95% CI: 0.051–0.585, *p* < 0.05), with predicted mean FDis of 3.08 (95% CI: 2.78–3.38) at low tide compared to higher tidal states. Time of day significantly affected functional evenness, with morning assemblages exhibiting higher evenness (*β* = 0.305, 95% CI: 0.042–0.569, *p* < 0.05; predicted mean: 0.95, 95% CI: 0.75–1.15) compared to evening observations (Appendix E: Table E4).

## 4 Discussion

### 4.1 Urbanization Disrupts Trait‐Based Assembly Through Environmental Filtering and Resource Homogenization

The pronounced interaction between the shoreline condition and season reveals fundamentally different community assembly processes operating along the urbanization gradient. Urban coastal systems function as environmental filters that select for broad‐niched generalists while excluding specialists, a pattern consistent with urban ecology theory (McKinney 2006; Sol et al. 2017) but previously undocumented in tropical African coastal systems. The year‐round dominance of large‐bodied generalist species, Pied Crow ( *C. albus* ) and Yellow‐billed Kite ( *M. aegyptius* ), in urbanized sites reflects several reinforcing mechanisms. Anthropogenic food subsidies from refuse and human waste create temporally stable resource landscapes that decouple community structure from natural phenological cycles (Oro et al. 2013). Structural simplification eliminates the microhabitat heterogeneity required by specialists: natural shorelines provide diverse substrates for foraging (mudflats, vegetation interfaces, riparian zones), whereas hardened urban shorelines offer homogeneous concrete and riprap (Lepczyk et al. 2017). Human disturbance (noise), foot traffic, and domestic predators directly exclude sensitive species, further favoring disturbance‐tolerant generalists (Sulemana et al. 2022).

In Ghana's rapidly urbanizing coastal zone, these pressures are particularly acute. Accra's coastline has experienced extensive hardening and infilling, displacing traditional wetland margins and mangrove fringes that support diverse waterbird assemblages. Alarmingly, some sites classified as “natural” during our study period have since undergone riprap installation and structural modification, exemplifying the rapid pace of coastal transformation occurring in real time. This ongoing habitat conversion highlights both the urgency of documenting baseline functional diversity and the ephemeral nature of remaining natural shoreline fragments (Aarif et al. 2025). The persistence of specialist‐dominated assemblages in remaining natural shoreline fragments highlights their irreplaceable conservation value, particularly for supporting seasonal migrants. The Common Sandpipers ( *A. hypoleucos* ) nearly 10‐fold increase in abundance during the dry season and the Black‐winged Stilt's ( *H. himantopus* ) exclusive appearance at natural sites exemplify how even small patches of intact habitat provide critical stopover resources for Palearctic migrants (Murray et al. 2014).

The seasonal turnover observed at natural shorelines, contrasting sharply with urban compositional stasis, demonstrates that undisturbed habitats maintain ecological responsiveness to temporal environmental variation. This responsiveness is not merely a descriptive pattern but a functional attribute linked to ecosystem resilience. Communities capable of seasonal reorganization can track resource fluctuations, partition niches temporally, and buffer against environmental agitations (Angeler and Allen 2016). Urban systems, by suppressing this temporal dynamism, may exhibit reduced capacity to respond to environmental change, a form of ecological inertia in which dominant generalists persist regardless of seasonal context (Grimm et al. 2008). Ecological inertia describes a state where community composition becomes decoupled from environmental variation, reducing the community's ability to track resource fluctuations or reorganize in response to disturbance (Sundstrom et al. 2017). This loss of responsiveness has critical implications for ecosystem resilience: seasonal turnover in functional traits allows communities to exploit different resource states throughout the year, buffering against environmental variability through temporal complementarity (Loreau et al. 2001). When urbanization eliminates this seasonal reorganization, communities lose their capacity for dynamic adjustment, becoming locked into a single functional configuration regardless of changing conditions (Angeler and Allen 2016).

This functional rigidity compromises ecosystem services that depend on seasonal trait turnover. Migratory shorebirds provide nutrient subsidies through guano deposition during the dry season, while resident insectivores peak in pest control during the wet season insect emergence; frugivores track seasonal fruiting patterns to provide seed dispersal services (Şekercioğlu et al. 2004; Wenny et al. 2011). Urban coastal sites, dominated year‐round by generalist scavengers and granivores, cannot deliver these seasonally specialized services, potentially disrupting nutrient cycling, pest regulation, and plant regeneration dynamics (Lepczyk et al. 2008; Wenny et al. 2011; Seress and Liker 2015). Moreover, reduced seasonal functional turnover may signal diminished capacity to respond to future environmental shifts, including climate‐driven changes in rainfall patterns or resource phenology (Angeler and Allen 2016). Natural habitats that maintain strong seasonal responses may therefore represent critical refugia not only for biodiversity but for ecosystem function and adaptive capacity in an era of rapid environmental change.

### 4.2 Seasonal Niche Partitioning and the Role of Migratory Influx

**FIGURE 6:**
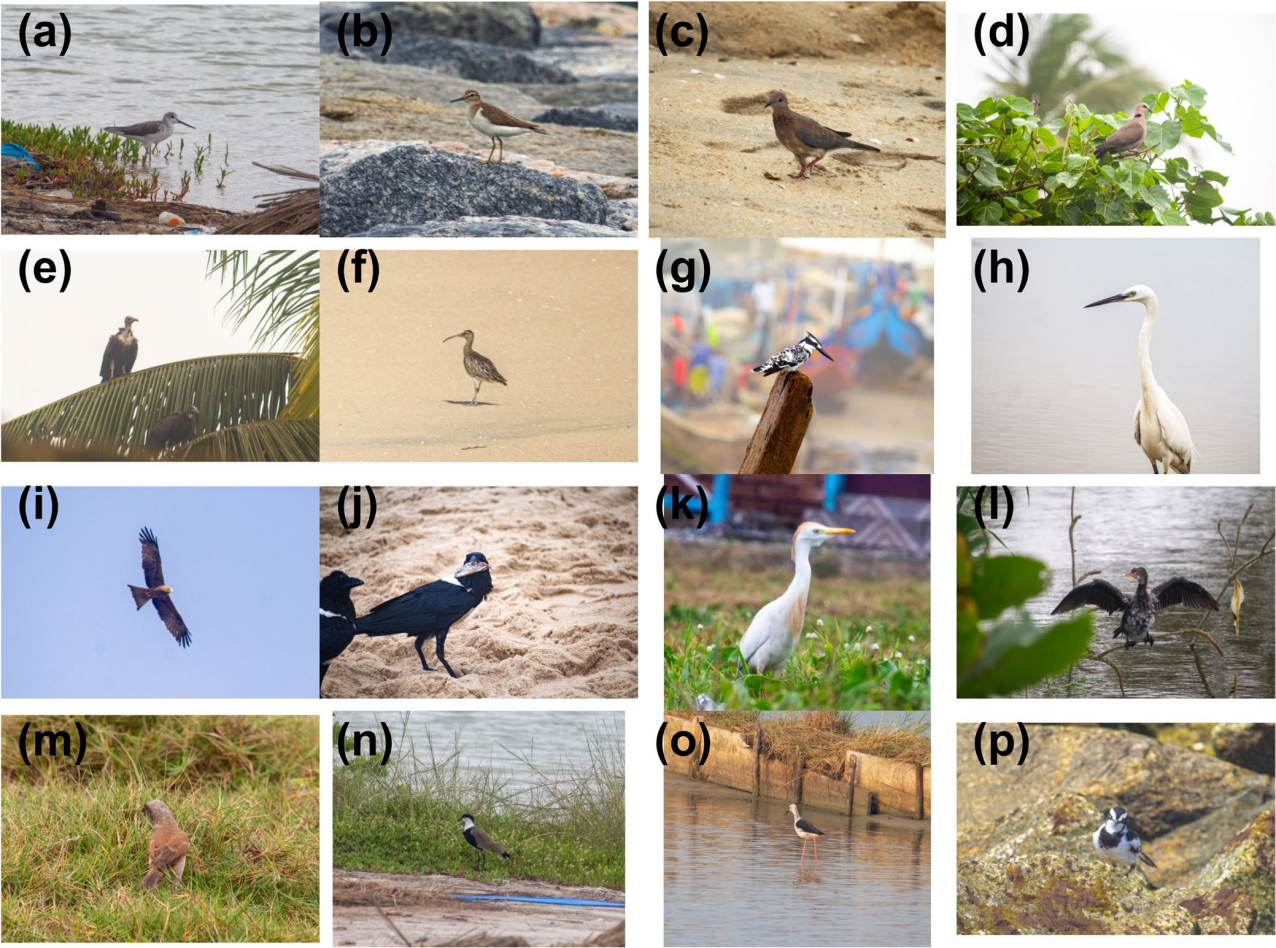
Representative bird species from contrasting habitat types along the Ghanaian coast. (a) Common Greenshank ( *Tringa nebularia* )—Palearctic visitor; (b) Common Sandpiper ( *Actitis hypoleucos* )—Palearctic visitor; (c) Laughing Dove ( *Spilopelia senegalensis* )—Resident; (d) Red‐eyed Dove ( *Streptopelia semitorquata* )—Resident; (e) Hooded Vulture ( *Necrosyrtes monachus* )—Resident; (f) Whimbrel ( *Numenius phaeopus* )—Palearctic visitor; (g) Pied Kingfisher ( *Ceryle rudis* )—Resident; (h) Western Reef Heron ( *Egretta gularis* )—Resident; (i) Yellow‐billed Kite ( *Milvus aegyptius* )—Intra‐African migrant; (j) Pied Crow ( *Corvus albus* )—Resident; (k) Cattle Egret ( *Bubulcus ibis* )—Resident; (l) Long‐tailed Cormorant ( *Microcarbo africanus* )—Resident; (m) Northern Gray‐headed Sparrow ( *Passer griseus* )—Resident; (n) Spur‐winged Lapwing ( *Vanellus spinosus* )—Resident; (o) Black‐winged Stilt ( *Himantopus himantopus* )—[Palearctic visitor OR Resident—depending on the observation season]; (p) African Pied Wagtail ( *Motacilla aguimp* )—Resident. Species represent diverse functional groups, including resident and migratory taxa, water‐associated and terrestrial species, and a range of foraging strategies and body sizes. Photographs by Samuel Etornam Tamkloe.

The strong seasonal differentiation in functional diversity reflects the ecological complexity of tropical coastal systems, where resident and migratory populations co‐occur within spatially constrained habitats. Our findings demonstrate that seasonal shifts are not merely changes in species numbers but fundamental reorganizations of functional trait space. The dry season expansion of functional dispersion likely reflects multiple interacting processes. Seasonal migratory influxes augment resident coastal assemblages with taxa possessing specialized foraging adaptations distinct from local guilds. Palearctic visitors such as the Whimbrel ( *Numenius phaeopus* ), Common Greenshank ( *Tringa nebularia* ), and Common Sandpiper ( *A. hypoleucos* ) intensively exploit intertidal mudflats. These periodic arrivals enrich the resident community, represented by species such as the Pied Kingfisher ( *Ceryle rudis* ), Western Reef Heron ( *Egretta gularis* ), and Cattle Egret ( *Bubulcus ibis* ), thereby increasing the diversity of ecological functions within the coastal habitats (Figure 6). However, these seasonal patterns coincide with marked shifts in resource availability: as the dry season progresses, invertebrates concentrate along shorelines (Piersma and Ntiamoa‐Baidu 1995), fish become more accessible in shrinking water bodies (Ntiamoa‐Baidu 1991), and receding water tables expose broad mudflat foraging grounds (Zwarts et al. 2016). Simultaneously, reduced vegetative cover increases detectability and enhances foraging efficiency for visual predators (Mokross et al. 2014).

Wet season contraction of functional dispersion coupled with increased divergence suggests environmental filtering toward functional extremes, though the mechanisms extend beyond the vegetation structure alone. Heavy vegetation during the wet season may exclude species dependent on open foraging substrates while favoring those adapted to dense cover (foliage gleaners) or those capable of exploiting flooded habitats (waders in ephemeral wetlands). Simultaneously, the wet season peak in insect abundance (particularly, termite emergences and dipteran swarms) supports aerial insectivores and opportunistic foragers (Holbech 2009), while seasonal flooding creates ephemeral wetlands that concentrate wading birds but exclude terrestrial foragers (Ntiamoa‐Baidu 1991). Functional divergence increases because intermediate trait combinations (e.g., mid‐sized generalists) experience reduced success under these contrasting conditions, while extremes (specialized insectivores, large‐bodied scavengers) partition the available niche space (Cornwell et al. 2006). West African coastal systems exhibit particularly pronounced resource pulses tied to both rainfall and marine productivity cycles, with nearshore fish aggregations during upwelling periods attracting piscivorous species (Camphuysen and Van Der Meer 2005) and coastal strand vegetation supporting granivores during seed maturation (Gatter 1999; Holbech 2009).

### 4.3 Weather‐Mediated Filtering Operates at Fine Temporal Scales

The prevalent association of cloud cover with multiple functional diversity metrics reveals that environmental filtering operates not only at seasonal timescales but at hourly and daily intervals. The simultaneous reduction in functional dispersion and increase in functional divergence under cloudy conditions illustrates how short‐term weather variation restructures active assemblages through differential detectability and activity patterns.

Overcast conditions disproportionately reduce the activity of species dependent on visual foraging and thermal soaring, swifts tracking aerial insects, raptors exploiting updrafts, and bee‐eaters hunting from exposed perches (Škrábal et al. 2023). This contracts overall trait space while leaving active species that are functionally extreme: ground‐foraging scavengers are less dependent on visual cues, and specialized insectivores exploit reduced competition during low‐activity periods. The result is a reduced but more divergent assemblage, demonstrating that functional diversity metrics capture orthogonal aspects of the community structure (Villéger et al. 2008).

Temperature and tidal influences further highlight how dynamic physical conditions shape functional diversity at fine scales. Soaring birds, including Yellow‐billed Kite and various raptors, actively exploit coastal areas, swooping to capture fish at the water surface or scavenging along shorelines (Ramos et al. 2023). Their activity is highly temperature‐dependent, requiring thermal development for energy‐efficient flight. Tidal exposure of mudflats creates ephemeral foraging opportunities that wading birds exploit during narrow temporal windows, demonstrating tight coupling between physical dynamics and functional diversity.

These microclimate associations suggest that functional diversity is continuously responsive to environmental variation across temporal scales, from hourly weather fluctuations to seasonal cycles to interannual climate variation. While our study provides a temporal snapshot insufficient for climate change inference, the demonstrated sensitivity to temperature and cloud cover implies that projected shifts in West African climate regimes (altered rainfall patterns, warming temperatures) could substantially restructure coastal bird communities (Massimino et al. 2020; Kim et al. 2022). Long‐term monitoring integrating functional trait approaches will be essential for detecting and understanding climate‐driven community shifts in tropical coastal systems.

### 4.4 Limitations and Future Directions

Several limitations warrant consideration. Our trait matrix, while encompassing diet, foraging mode, and morphology, could be expanded to include behavioral traits (boldness, disturbance tolerance) and physiological traits (thermal tolerance). Future studies should employ community‐weighted mean trait analyses to identify specific trait shifts along disturbance gradients, complementing the multivariate functional diversity approach used here. Functional beta diversity approaches would quantify whether sites are becoming more similar in trait composition, directly testing homogenization hypotheses that cannot be evaluated with alpha diversity indices alone. Temporal coverage represents another limitation. Our study spanned major seasonal contrasts (dry vs. wet) but did not capture interannual variation or multi‐year dynamics. Long‐term datasets are particularly scarce for African coastal bird communities (Lee et al. 2021), but they are essential for disentangling climate effects from stochastic variation. Finally, linkages between functional diversity and ecosystem functioning remain underexplored in tropical coastal systems. Do the functional shifts we document translate to altered ecosystem services (seed dispersal, pest suppression, nutrient cycling)? Addressing this question requires integrating functional trait data with ecosystem process measurements. Despite these limitations, our work provides a baseline insight into functional diversity dynamics in tropical West African coastal bird communities, demonstrating that urbanization disrupts not only community composition but the fundamental temporal dynamics that characterize natural systems.

## 5 Conclusion and Recommendations

Tropical coastal systems face unprecedented development pressure; however, conservation strategies often focus narrowly on species richness while overlooking the functional roles species play and how these roles vary across space and time. This study demonstrates that urbanization restructures avian functional diversity through multiple pathways: filtering communities toward generalist species, suppressing seasonal dynamics, and reducing ecosystem responsiveness to environmental variation. Natural shoreline habitats are not merely species‐rich alternatives to urban areas; they are functionally distinct systems that maintain temporal complementarity and ecological responsiveness essential for long‐term resilience.

Our findings have direct implications for coastal conservation planning in rapidly developing tropical regions. Preserving the remaining natural shoreline fragments should be prioritized not only for supporting high species richness but also for maintaining the seasonal functional turnover that supports ecosystem stability. We recommend the following three conservation actions for Ghana and similar West African coastal systems: (1) prioritize the legal protection of remaining natural shoreline segments, which support the highest functional diversity and seasonal turnover; (2) implement policies that restrict further hard armoring (riprap and seawall construction) along functionally rich stretches, particularly those identified as supporting diverse foraging guilds and migratory species; and (3) mandate the incorporation of seasonal bird surveys and functional diversity metrics into coastal development Environmental Impact Assessments (EIAs), ensuring that temporal dynamics are considered alongside traditional species richness assessments.

The fact that some of our study sites have already been modified with riprap installations since data collection highlights the alarming rate of coastal transformation and the narrow window available for conservation action. Coastal management must move beyond static species inventories toward dynamic, trait‐based approaches that recognize temporal variability as an essential component of biodiversity. In Ghana and throughout West Africa, where coastal urbanization is accelerating, immediate action is needed to protect undisturbed shoreline habitats before functional erosion becomes irreversible. More broadly, this work contributes to the understanding of how anthropogenic change operates in tropical systems, regions that harbor extraordinary biodiversity yet remain underrepresented in ecological theory.

## Author Contributions

**Samuel E. Tamekloe:** conceptualization (lead), data curation (lead), formal analysis (lead), funding acquisition (lead), investigation (lead), methodology (lead), visualization (lead), writing – original draft (lead), writing – review and editing (lead). **Joseph K. Daniels:** formal analysis (supporting), visualization (supporting), writing – original draft (supporting), writing – review and editing (supporting). **Kweku A. Monney:** conceptualization (supporting), supervision (supporting), writing – original draft (supporting), writing – review and editing (supporting). **Justus P. Deikumah:** conceptualization (lead), methodology (supporting), supervision (lead), writing – original draft (supporting), writing – review and editing (supporting).

## Funding

This study was funded by the A.P. Leventis Ornithological Research Institute (APLORI).

## Ethics Statement

No approval of research ethics committees was required to accomplish the goals of this study because the work was conducted entirely in the field and did not require trapping of any bird species.

## Conflicts of Interest

The authors declare no conflicts of interest.

## Data Availability

The data analyzed in this study were collected by the authors and organized in Microsoft Excel 2019 spreadsheets. Data analysis was conducted using R version 4.5.1 and associated open‐source packages. All datasets and fully annotated R code are publicly available on the Figshare data repository at https://doi.org/10.6084/m9.figshare.29328716. The repository includes the complete dataset (CSV format), data summary (Excel format), and analysis code (R file). The full trait dataset is available in the repository as spreadsheet files to facilitate accessibility and potential reuse by other researchers.

## Appendix A Data Distribution

**TABLE A1:** Shapiro–wilk normality tests (*p* < 0.05 suggests non‐normal).

Metric	W_statistic	*p*	Distribution	Signif
FRic	0.9580	1e‐07	Non‐normal	***
FEve	0.9444	0e+00	Non‐normal	***
FDiv	0.9586	2e‐07	Non‐normal	***
FDis	0.9384	0e+00	Non‐normal	***

*Note:* Significance levels are indicated as follows: *** *p* < 0.001.

## Appendix B Seasonal Variation in Bird Functional Diversity

**TABLE B1:** Kruskal–Wallis per FD metric (season).

FD_metric	*p*	Signif
FDis	0.0000000	***
FDiv	0.0221348	*
FEve	0.8225676	ns
FRic	0.0265686	*

*Note:* Significance levels are indicated as follows: *** *p* < 0.001; * *p* < 0.05; ns = not significant (*p* > 0.05).

**TABLE B2:** Pairwise Dunn Tests for significant metrics (season).

FD_metric	Comparison	*Z*	P.adj	Signif
FDis	Dry—wet	5.709	0.0000	***
FDiv	Dry—wet	−2.288	0.0221	*
FRic	Dry—wet	−2.218	0.0266	*

*Note:* Significance levels are indicated as follows: *** *p* < 0.001; * *p* < 0.05.

**TABLE B3:** Descriptive statistics: Mean ± SE and 95% confidence intervals (season).

Season	FD metric	*n*	Mean	SD	SE	CI_lower	CI_upper	Median	Min	Max
Dry	FDis	78	3.6365	1.4179	0.1605	3.3168	3.9562	3.1743	1.2537	8.9073
Dry	FDiv	78	0.8775	0.0655	0.0074	0.8627	0.8923	0.8849	0.7333	0.9938
Dry	FEve	78	0.6986	0.1694	0.0192	0.6604	0.7368	0.7479	0.2884	1.0000
Dry	FRic	78	5.2894	4.3030	0.4872	4.3192	6.2596	5.0886	0.0000	13.2195
Wet	FDis	71	2.3942	1.0256	0.1217	2.1515	2.6370	2.3940	0.3941	5.2197
Wet	FDiv	71	0.9008	0.0728	0.0086	0.8836	0.9181	0.9123	0.7441	0.9980
Wet	FEve	71	0.6961	0.1997	0.0237	0.6489	0.7434	0.7296	0.0774	0.9949
Wet	FRic	71	6.7248	3.2745	0.3886	5.9497	7.4999	7.0679	0.0682	16.5804

## Appendix C Shoreline Categorization Association With Bird Functional Diversity

**TABLE C1:** Kruskal–Wallis per FD metric (shoreline state).

FD_metric	*p*	Signif
FDis	0.0656580	ns
FDiv	0.0996842	ns
FEve	0.0021832	**
FRic	0.0238190	*

*Note:* Significance levels are indicated as follows: ** *p* < 0.01; * *p* < 0.05; ns = not significant (*p* > 0.05).

**TABLE C2:** Pairwise Dunn tests for significant metrics (shoreline state).

FD_metric	Comparison	*Z*	P.adj	Signif
FEve	Natural—urbanized	−3.064	0.00218	**
FRic	Natural—urbanized	−2.260	0.02380	*

*Note:* Significance levels are indicated as follows: ** *p* < 0.001; * *p* < 0.05.

**TABLE C3:** Descriptive statistics: Mean ± SE and 95% confidence intervals (shoreline state).

Shoreline state	FD metric	*n*	Mean	SD	SE	CI_lower	CI_upper	Median	Min	Max
Natural	FDis	68	2.9536	1.7348	0.2104	2.5337	3.3735	2.6879	0.3941	8.9073
Natural	FDiv	68	0.8965	0.0768	0.0093	0.8779	0.9151	0.9086	0.7333	0.9980
Natural	FEve	68	0.6535	0.1889	0.0229	0.6078	0.6993	0.6735	0.0774	0.9864
Natural	FRic	68	5.2274	3.9042	0.4735	4.2824	6.1725	5.6674	0.0000	13.5688
Urbanized	FDis	81	3.1209	1.0194	0.1133	2.8955	3.3463	2.9577	1.1794	6.5861
Urbanized	FDiv	81	0.8820	0.0632	0.0070	0.8680	0.8960	0.8915	0.7441	0.9927
Urbanized	FEve	81	0.7343	0.1722	0.0191	0.6962	0.7724	0.7950	0.2884	1.0000
Urbanized	FRic	81	6.5996	3.8111	0.4235	5.7569	7.4423	6.9876	0.0005	16.5804

## Appendix D Interactive Relationship Between the Season and Shoreline Categories and Bird Functional Diversity

**TABLE D1:** Kruskal–Wallis per FD metric (season × shoreline state).

FD_metric	*p*	Signif
FDis	0.0000000	***
FDiv	0.0002467	***
FEve	0.0191984	*
FRic	0.0125932	*

*Note:* Significance levels are indicated as follows: *** *p* < 0.001; * *p* < 0.05.

**TABLE D2:** Pairwise Dunn tests for significant metrics (season × shoreline state).

FD_metric	Comparison	*Z*	P.adj	Signif
FDis	Natural_Dry—Natural_Wet	6.1622035	0.00E+00	***
FDis	Natural_Wet—Urbanized_Dry	−5.4856261	2.00E‐07	***
FDis	Natural_Dry—Urbanized_Wet	2.8850524	2.35E‐02	*
FDis	Natural_Wet—Urbanized_Wet	−3.4059916	3.96E‐03	**
FDiv	Natural_Dry—Natural_Wet	−4.0069777	3.69E‐04	***
FDiv	Natural_Wet—Urbanized_Dry	3.0148898	1.54E‐02	*
FDiv	Natural_Wet—Urbanized_Wet	3.5270194	2.52E‐03	**
FRic	Natural_Dry—Urbanized_Wet	−3.253795	6.83E‐03	**

*Note:* Significance levels are indicated as follows: *** *p* < 0.001; ** *p* < 0.01; * *p* < 0.05.

**TABLE D3:** Descriptive statistics: Mean ± SE and 95% confidence intervals (season × shoreline state).

Season	Shoreline state	FD_metric	*n*	Mean	SD	SE	CI_lower	CI_upper
Dry	Natural	FDis	34	4.0069	1.7055	0.2925	3.4118	4.6019
Dry	Natural	FDiv	34	0.8631	0.0745	0.0128	0.8372	0.8891
Dry	Natural	FEve	34	0.669	0.1525	0.0262	0.6158	0.7223
Dry	Natural	FRic	34	4.264	4.4274	0.7593	2.7192	5.8087
Dry	Urbanized	FDis	44	3.3503	1.0843	0.1635	3.0207	3.68
Dry	Urbanized	FDiv	44	0.8886	0.0561	0.0085	0.8715	0.9057
Dry	Urbanized	FEve	44	0.7215	0.1797	0.0271	0.6668	0.7761
Dry	Urbanized	FRic	44	6.0818	4.0791	0.6149	4.8417	7.3219
Wet	Natural	FDis	34	1.9003	0.9566	0.1641	1.5665	2.234
Wet	Natural	FDiv	34	0.9299	0.0643	0.011	0.9074	0.9523
Wet	Natural	FEve	34	0.6381	0.2206	0.0378	0.5611	0.715
Wet	Natural	FRic	34	6.1909	3.0713	0.5267	5.1193	7.2626
Wet	Urbanized	FDis	37	2.8481	0.8741	0.1437	2.5567	3.1396
Wet	Urbanized	FDiv	37	0.8741	0.0707	0.0116	0.8505	0.8977
Wet	Urbanized	FEve	37	0.7495	0.1639	0.027	0.6949	0.8042
Wet	Urbanized	FRic	37	7.2154	3.4185	0.562	6.0756	8.3551

**TABLE D4:** Top 15 species: Natural shorelines (dry vs. wet season side‐by‐side).

Rank	Dry season			Wet season		
	Species	Family	Count	Species	Family	Count
1	Village Weaver	Ploceidae	625	Village Weaver	Ploceidae	1205
2	Yellow‐billed Kite	Accipitridae	385	Little Swift	Apodidae	149
3	Pied Crow	Corvidae	267	Pied Crow	Corvidae	121
4	Little Swift	Apodidae	164	Yellow‐Billed Kite	Accipitridae	46
5	Common Sandpiper	Scolopacidae	55	Long‐Tailed Cormorant	Phalacrocoracidae	36
6	Black‐winged Stilt	Recurvirostridae	53	Cattle Egret	Ardeidae	23
7	Ethiopian Swallow	Hirundinidae	51	Western Reef Heron	Ardeidae	22
8	Bronze Mannikin	Estrildidae	44	Pied Kingfisher	Alcedinidae	16
9	Pied Kingfisher	Alcedinidae	41	Laughing Dove	Columbidae	14
10	Long‐Tailed Cormorant	Phalacrocoracidae	31	Bronze Mannikin	Estrildidae	13
11	Western‐Reef Heron	Ardeidae	30	Northern Gray‐headed Sparrow	Passeridae	10
12	Cattle Egret	Ardeidae	27	African Pied Wagtail	Motacillidae	7
13	Eurasian Curlew	Scolopacidae	27	Common Bulbul	Pycnonotidae	6
14	Laughing Dove	Columbidae	23	Common Sandpiper	Scolopacidae	6
15	Common Bulbul	Pycnonotidae	16	Common Tern	Laridae	6

**TABLE D5:** Top 15 species: Urbanized shorelines (dry vs. wet season side‐by‐side).

Rank	Dry season			Wet season		
	Species	Family	Count	Species	Family	Count
1	Pied Crow	Corvidae	578	Pied Crow	Corvidae	213
2	Yellow‐Billed Kite	Accipitridae	498	Village Weaver	Ploceidae	120
3	Little Swift	Apodidae	127	Yellow‐Billed Kite	Accipitridae	114
4	Village Weaver	Ploceidae	90	Long‐Tailed Cormorant	Phalacrocoracidae	88
5	Long‐Tailed Cormorant	Phalacrocoracidae	86	Little Swift	Apodidae	85
6	African Palm Swift	Apodidae	58	Western‐Reef Heron	Ardeidae	26
7	Cattle Egret	Ardeidae	58	Laughing Dove	Columbidae	24
8	Pied Kingfisher	Alcedinidae	46	pied Kingfisher	Alcedinidae	22
9	Hooded Vulture	Accipitridae	42	Hooded Vulture	Accipitridae	21
10	African Pied Wagtail	Motacillidae	34	Common Bulbul	Pycnonotidae	16
11	Northern Gray‐Headed Sparrow	Passeridae	33	Cattle Egret	Ardeidae	13
12	Ethiopian Swallow	Hirundinidae	31	Northern Gray‐Headed Sparrow	Passeridae	11
13	Laughing Dove	Columbidae	30	African Palm Swift	Apodidae	9
14	Curlew Sandpiper	Scolopacidae	24	Common Sandpiper	Scolopacidae	9
15	Common Bulbul	Pycnonotidae	23	Ethiopian Swallow	Hirundinidae	8

## Appendix E GLMM Diagnostic Plots, Multicollinearity, Spatial Autocorrelation Checks, and Model Summaries

**TABLE E1:** Moran's *I* Test for spatial autocorrelation (*p* > 0.05 = no significant autocorrelation).

Model	Morans_*I*	Expectation	Variance	*p*	Autocorrelation	Signif
FRic	−1.5309	−0.0559	−0.003333	0.937	None	ns
FEve	−1.1580	−0.0431	−0.003333	0.877	None	ns
FDiv	0.1313	0.0012	−0.003333	0.448	None	ns
FDis	−0.4777	−0.0197	−0.003333	0.684	None	ns

*Note:* Significance levels are indicated as follows: ns = not significant (*p* > 0.05).

**TABLE E2:** Variance inflation factors (VIF < 5: Low multicollinearity; VIF > 10: High concern).

Variable	VIF	Interpretation	Multicollinearity
Wind speed	3.267	Low	No concern
Air temperature	2.570	Low	No concern
Time of day	1.953	Low	No concern
Cloud cover	1.399	Low	No concern
Tidal level	1.120	Low	No concern
Humidity	1.062	Low	No concern

**TABLE E3:** Summary of generalized linear mixed models (GLMMs) for functional diversity metrics.

Metric	Predictor	*β* (estimate)	95% CI (low)	95% CI (high)	SE	*p*	R2m	R2c
FRic	Intercept	20.073	4.904	35.242	7.739	0.0095	0.037	0.310
	Humidity	−0.021	−0.058	0.016	0.019	0.2727	0.037	0.310
	Cloud cover	0.014	−0.001	0.029	0.008	0.0656	0.037	0.310
	Wind speed	−0.030	−0.130	0.071	0.051	0.5641	0.037	0.310
	Air temperature	−0.446	−0.977	0.085	0.271	0.0996	0.037	0.310
	Tidal level (low)	−0.543	−1.419	0.333	0.447	0.2243	0.037	0.310
	Time of day (morning)	−0.594	−1.707	0.518	0.568	0.2953	0.037	0.310
FEve	Intercept	1.134	−2.484	4.753	1.846	0.5390	0.132	0.717
	Humidity	0.007	−0.002	0.015	0.004	0.1239	0.132	0.717
	Cloud cover	−0.004	−0.007	0.000	0.002	0.0410	0.132	0.717
	Wind speed	0.017	−0.007	0.041	0.012	0.1652	0.132	0.717
	Air temperature	−0.050	−0.176	0.077	0.065	0.4424	0.132	0.717
	Tidal level (low)	0.080	−0.127	0.286	0.105	0.4497	0.132	0.717
	Time of day (morning)	0.305	0.042	0.569	0.134	0.0231	0.132	0.717
FDiv	Intercept	3.956	0.889	7.023	1.565	0.0115	0.169	0.961
	Humidity	−0.003	−0.011	0.005	0.004	0.4117	0.169	0.961
	Cloud cover	0.004	0.002	0.007	0.002	0.0030	0.169	0.961
	Wind speed	−0.007	−0.027	0.013	0.010	0.4857	0.169	0.961
	Air temperature	−0.056	−0.163	0.050	0.054	0.2996	0.169	0.961
	Tidal level (low)	−0.005	−0.174	0.164	0.086	0.9550	0.169	0.961
	Time of day (morning)	−0.182	−0.402	0.037	0.112	0.1029	0.169	0.961
FDis	Intercept	−5.214	−9.849	−0.579	2.365	0.0275	0.197	0.469
	Humidity	0.003	−0.008	0.014	0.006	0.6102	0.197	0.469
	Cloud cover	−0.012	−0.017	−0.008	0.002	< 0.0001	0.197	0.469
	Wind speed	−0.001	−0.032	0.030	0.016	0.9394	0.197	0.469
	Air temperature	0.316	0.153	0.478	0.083	0.0001	0.197	0.469
	Tidal level (low)	0.318	0.051	0.585	0.136	0.0196	0.197	0.469
	Time of day (morning)	0.128	−0.213	0.468	0.174	0.4624	0.197	0.469

**TABLE E4:** Significant categorical predictors with predicted means (95% CI).

Metric	Predictor	Estimate	CI_low	CI_high	SE	*p*	R2m	R2c	Predicted_Mean_CI
FEve	Time of day (morning)	0.305	0.042	0.569	0.134	0.0230	0.132	0.717	0.95 [0.75–1.15]
FDis	Tidal level (low)	0.318	0.051	0.585	0.136	0.0196	0.197	0.469	3.08 [2.78–3.38]
